# Facile Synthesis of Porous ZnO Nanoparticles Efficient for Photocatalytic Degradation of Biomass-Derived Bisphenol A Under Simulated Sunlight Irradiation

**DOI:** 10.3389/fbioe.2020.616780

**Published:** 2021-01-14

**Authors:** Yujie Wang, Kang Hu, Zhiyu Yang, Chenlu Ye, Xin Li, Kai Yan

**Affiliations:** ^1^Guangzhou Key Laboratory of Environmental Catalysis and Pollution Control, School of Environmental Science and Engineering, Institute of Environmental Health and Pollution Control, Guangdong University of Technology, Guangzhou, China; ^2^Guangdong Provincial Key Laboratory of Environmental Pollution Control and Remediation Technology, School of Environmental Science and Engineering, Sun Yat-sen University, Guangzhou, China; ^3^Ganjiang Innovation Academy, Chinese Academy of Sciences, Guangzhou, China

**Keywords:** photocatalytic degradation, BPA, porous ZnO, kinetics, mechanisms

## Abstract

Bisphenol A (BPA) produced from biomass is a typical endocrine disrupting compound that is carcinogenic and genotoxic and can be accumulated in water due to its extensive use and difficult degradation. In this study, the porous ZnO photocatalyst with core-shell structure and large surface area was successfully developed for the efficient photocatalytic degradation of BPA. The various effects of calcination temperatures, BPA concentrations, ZnO dosages, pH and inorganic ions on the degradation performance were systemically studied. The results showed that 99% degradation of BPA was achieved in 1 h using the porous ZnO calcined at 550°C under the conditions of 30 mg/L BPA, 1 g/L ZnO, and pH of 6.5. Besides, the inhibition effects of anions for the photocatalytic removal of BPA decreased in the order of H_2_${\text{PO}}_{4}^{-}$ > ${\text{HCO}}_{3}^{-}$ > ${\text{SO}}_{4}^{2-}$ > Cl^−^, while the cations K^+^, Ca^2+^, and Na^+^ had little effect on the photocatalytic degradation of BPA. The results of scavenging experiments showed that h^+^, ·${\text{O}}_{2}^{-}$, and e^−^ played the key role in the photocatalytic degradation process. Finally, the main pathways of BPA degradation were proposed based on ten intermediates found in the degradation process. This work may provide a good guideline to degrade various endocrine disrupting compounds in wastewater treatment.

## Introduction

Bisphenol A (BPA) derived from biomass is usually utilized as the stabilizing agent in the processing of plastics and epoxy resins. In 2015, around 7.7 million metric tons of BPA was consumed with an annual increase of almost 5% until 2022 all over the world (Ozyildiz et al., 2019; Wang et al., 2019). However, BPA is regarded as a typical endocrine disrupting compound (EDC) and is carcinogenic and genotoxic. 193 ng/L BPA was detected in the surface water and 39 ng/L in the subsurface and bottom waters (Rachna et al., 2019). The release of BPA into the environment poses a threat to human health even at low exposure levels (Selvakumar et al., 2019). As is well-known, BPA is stable in an aqueous solution and is refractory to degradation because it consists of two benzene rings in a symmetrical structure. Thus, it is very urgent and significant to develop effective processes for BPA removal from water (Ye et al., 2019; Zhang et al., 2019).

Photocatalysis has been considered to be one of the promising technologies for degradation of BPA (He et al., 2019a; Sabouni and Gomaa, 2019). Among various semiconductor metal oxides, ZnO is one of the most extensively studied photocatalysts used to degrade dyes such as acid violet (González-Casamachin et al., 2019), rhodamine B (Hao et al., 2019; Lops et al., 2019), reactive red (Rezk et al., 2019), etc. Nevertheless, some limitations such as ultraviolet light response and low quantization efficiency hinder its practical application. Recently, the strategy of doping has been carried out to enhance photocatalytic degradation activity of BPA (Meng et al., 2015; Vaiano et al., 2018). Bechambi et al. (2015c, 2016) showed that the Ce-[15] doped ZnO obtained the complete degradation of BPA after 24 h of UV irradiation. Bechambi et al. (2015b) also developed the C-[15]doped ZnO photocatalyst to promote the photodegradation of BPA. Kamaraj et al. (2014) doped Ce in ZnO to generate a sunlight-active photocatalyst which could degrade 98% 10 mg/L BPA in 8 h under sunlight in the summer. The photocatalytic degradation of BPA was also enhanced by the modification of ZnO with doping Ag (Jasso-Salcedo et al., 2014; Bechambi et al., 2015a).

As is well-known, the morphological features and surface area have a great influence on the photocatalytic activity (Dong et al., 2017; He et al., 2019b; Wetchakun et al., 2019). Fabrication of different morphological features of ZnO is another strategy to optimize the photocatalytic performance (Dong et al., 2020). The rational design of nanostructured ZnO with morphological features (such as: spherality, core-shell, nanowire and nanosheet etc.) and high effective surface area can make extraordinary progress in enhancing the activities for photocatalysis applications (Theerthagiri et al., 2019). Taylor et al. (2019) synthesized ZnO nanowire and reported the enhanced photo-corrosion resistance, the improved photo-response, and stability. Qi et al. (2013) fabricated ZnO nanoflower to eliminate methyl orange which exhibited higher photocatalytic activity than ZnO fragments. Our previous works also showed that the mesoporous structure was beneficial for the photocatalytic performance (Hu et al., 2020; Li et al., 2020; Wang et al., 2020b).

Herein we reported the mesoporous ZnO photocatalyst with core-shell structure and large surface area which was controllably synthesized by a hydrothermal synthesis method with the aid of urea. So far, there is little investigation of the control of the structure of ZnO to improve the photocatalytic activity for photodegradation of BPA. In this study, the prepared ZnO photocatalyst showed better performance for the photodegradation of BPA than the ones reported in the previous literature. A combination analysis of XRD, XPS, SEM, TEM, and HR-TEM was conducted to investigate the physicochemical properties, morphology, and structure of the prepared ZnO. Furthermore, the various effects of calcination temperatures, BPA concentrations, ZnO dosages, pH, and inorganic ions on the degradation performance were systematically analyzed. Besides, scavenging experiments and electron spin resonance (ESR) technique were performed to investigate reaction mechanisms. The main pathways of BPA degradation were also rationally deduced based on the identified intermediates by LC-MS.

## Methods and Materials

### Chemicals

Zinc acetate dihydrate (99.99%), bisphenol A (99%), and hydrogen peroxide (30 wt.%) were purchased from Aladdin (Shanghai, China). Urea (99%), tert-butyl alcohol (99.5%), p-benzoquinone (99%), ammonium oxalate (98%), sodium hydroxide (97%) and dimethyl sulfoxide (99.7%) were obtained from Macklin (Shanghai, China). The other chemicals were at least analytical reagents and utilized directly without any purification.

### Synthesis of Porous ZnO

**Figure 1:**
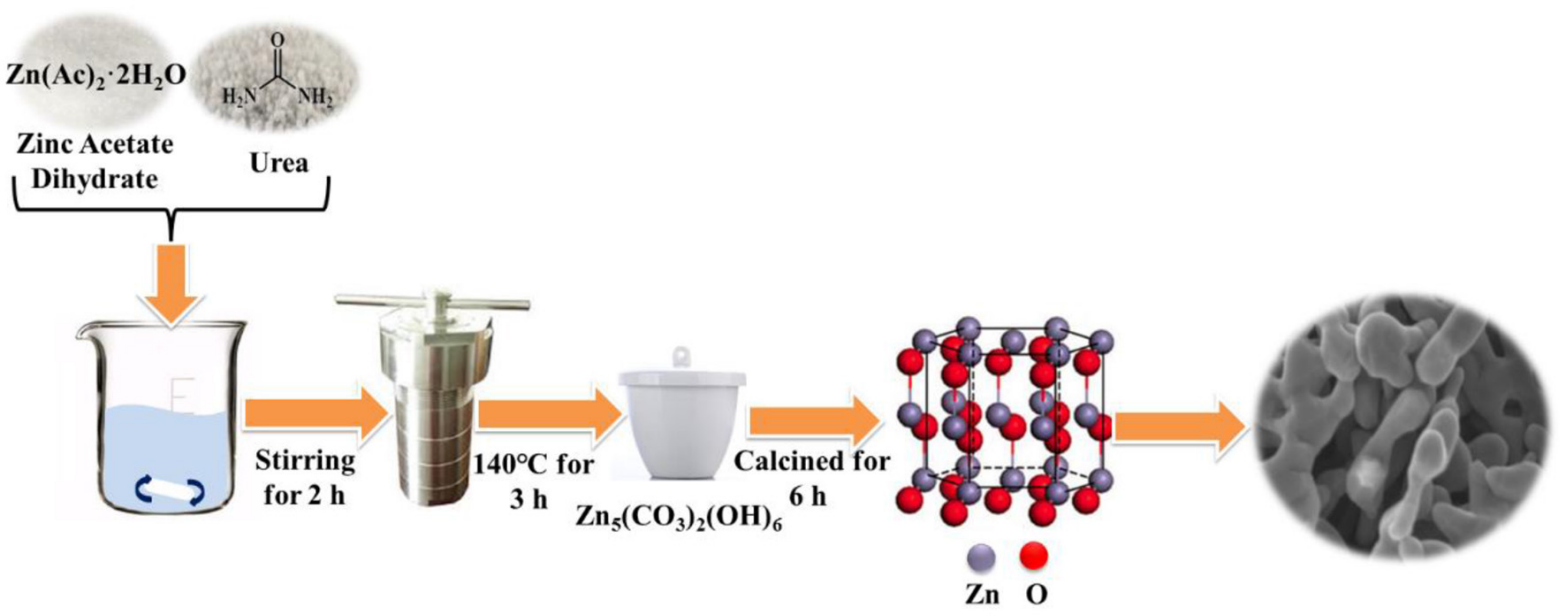
Schematic illustration of the synthesis of porous ZnO photocatalyst.

In a typical synthesis, 0.002 mol zinc acetate dihydrate and 0.02 mol urea were stirred for 2 h at room temperature under 400 rpm to dissolve in 40 mL deionized water. Then, the solution was transferred into a 100 mL Teflon-lined stainless autoclave which was kept at 140°C for 3 h and finally cooled naturally. The procedure adopted for the synthesis of porous ZnO photocatalyst was depicted in Figure 1. The sample obtained was washed with the deionized water and absolute ethanol for several times and then treated with centrifugation under 10,000 rpm in 5 min. After drying in air at 80°C for 12 h, the residual powder was calcined at various temperatures (350°, 450°, 550°, and 650°C) for 6 h with a heating rate of 3°C/min in air. Then, the combination analysis of XRD, XPS, SEM, TEM, and HR-TEM was conducted on the as-prepared samples. The detailed information is shown in Supporting Information (SI).

### BPA Photocatalytic Degradation Procedures

The photocatalytic performance tests of porous ZnO photocatalysts were investigated under a 300 W Xenon lamp (CEL-PF300-T8E, Beijing China Education Au-light Co., Ltd.), which was used to simulate sunlight irradiation. The as-prepared ZnO photocatalyst (50 mg) was added to 50 mL BPA solution. Then, the suspension was treated by ultrasound for 2 min and stirred for 30 min to reach adsorption equilibrium in the dark. At 15-min intervals, ~1.0 ml solution was withdrawn for analysis after the catalyst was removed through a 0.22 μm PTFE filter. The BPA concentration was detected using a high-performance liquid chromatography (HPLC, Shimadzu LC-20 A) with column temperature at 30°C. The mobile phase composition was ultrapure water/methanol (30/70, v/v). The flow rate was kept at 1 ml/min and the injection volume was 10 μl.

## Results and Discussion

### Characterization of the Photocatalysts

**Figure 2:**
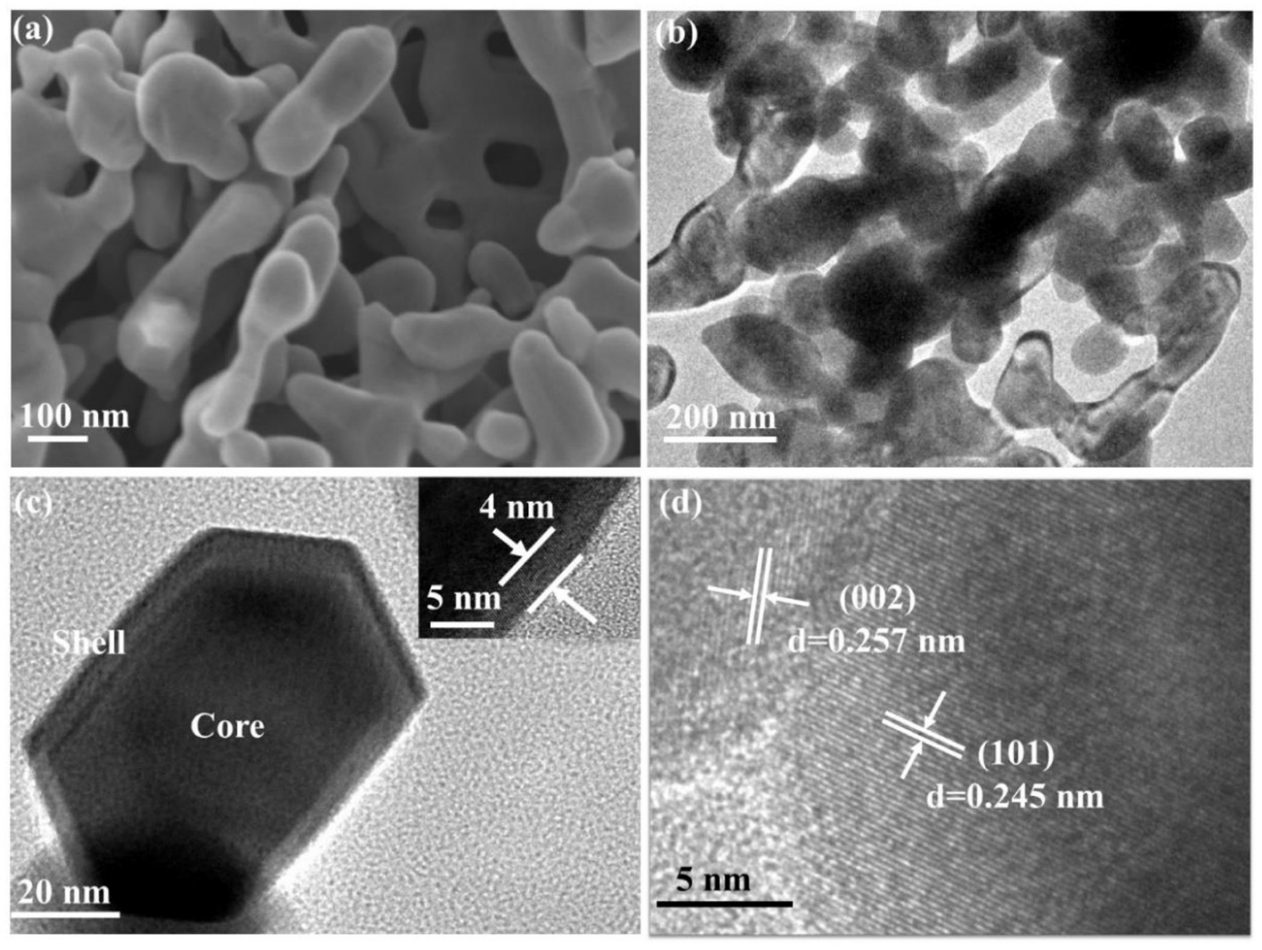
**(a)** SEM; **(b,c)** TEM (Inset was the shell thickness); **(d)** HR-TEM of porous ZnO calcined under 550°C.

The crystal phase and structure of the fabricated porous ZnO was analyzed by XRD (Supplementary Figure 1). Supplementary Figure 1A shows that the maximum relative intensity for porous ZnO were found at 31.8°, 34.4°, 36.2°, and 56.6° with d-spacing of 2.8143, 2.6033, 2.4759, and 1.6247, respectively. These corresponding peaks are, respectively, related to the (100), (002), (101), and (110) crystal planes, which are well-consistent with JCPDS No. 36-1451 (a = b = 3.250 Å, c = 5.207 Å), suggesting the hexagonal structure. Supplementary Figure 1B shows that the diffraction peaks of the precursor are well-consistent with Zn_5_(CO_3_)_2_(OH)_6_ (JCPDS No. 19-1458). After it was annealed at 550°C for 6 h, no other peak was detected, which indicated the precursor had completely transformed into the pure ZnO crystal. The details of the calcination process were described in SI. The general morphology and microstructure of the fabricated ZnO photocatalyst were further analyzed by SEM. Figure 2a and Supplementary Figure 2 display the SEM images of the as-prepared ZnO at various calcination temperatures. Supplementary Figures 2A,B shows the ZnO is form of plate structure with porosity at 350° and 450°C. Figures 2a,b and Supplementary Figure 2C exhibit the porous framework structure under the calcination temperatures of 550° and 650°C. These results revealed that the calcination temperature has a significant effect on the morphology and structure of ZnO. The detailed microstructure was further studied by TEM and HR-TEM (Figures 2c,d). Core-shell hexagonal crystal nanoparticles were clearly observed in the range of 20–50 nm in length. The shell thickness is ~4 nm (inset in Figure 2c). It can also be clearly seen that ZnO nanoparticles with lattice spacing of 0.245 nm and 0.257 nm correspond to (101) and (002) plane, respectively (Figure 2d).

**Figure 3:**
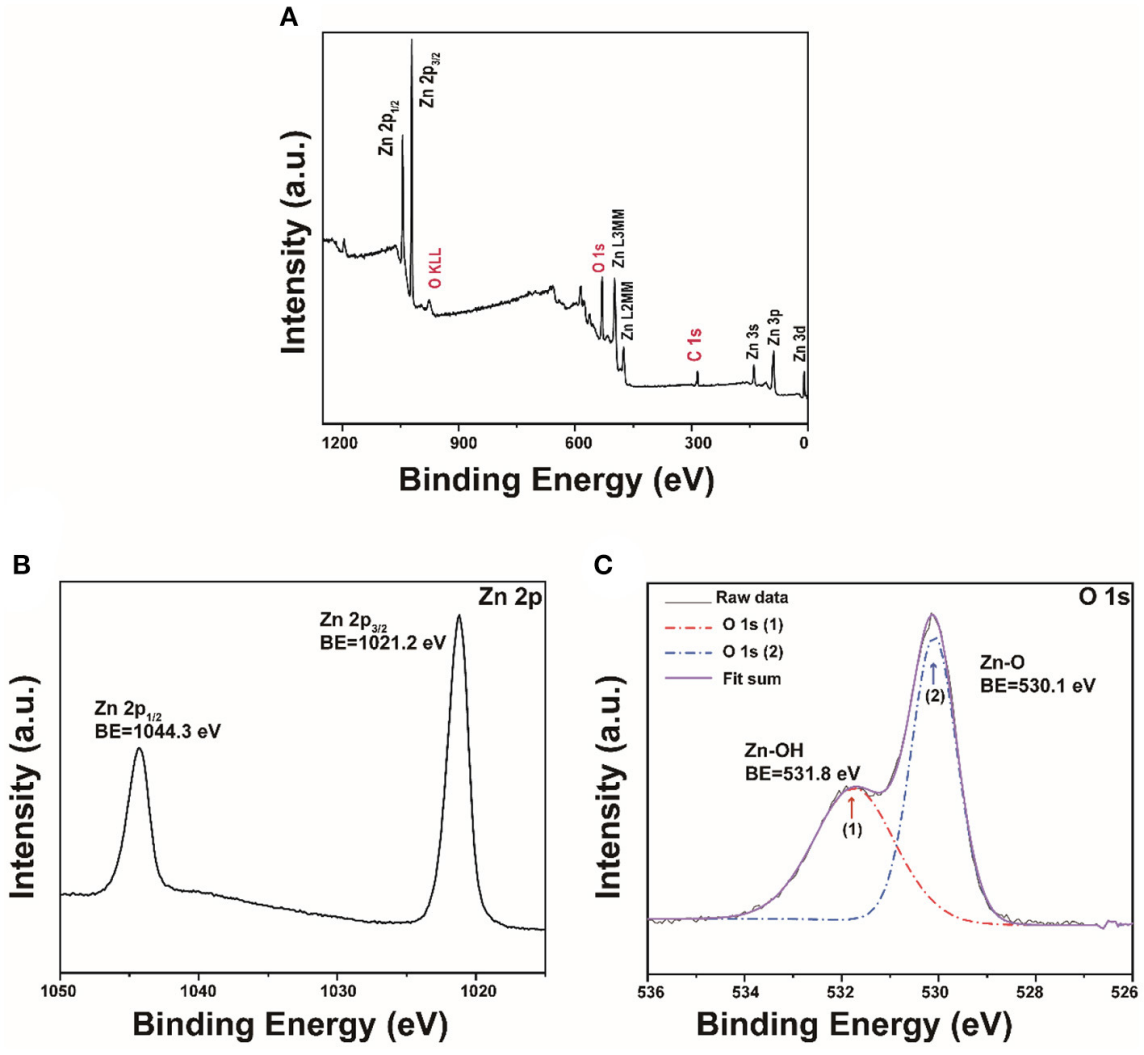
**(A)** XPS survey of the porous ZnO; The high-resolution XPS spectra: **(B)** Zn 2p and **(C)** O 1s.

XPS was then applied to analyze the chemical state of the synthesized ZnO. The result shows that the as-prepared nanoparticles are primarily composed of Zn, O, and C (Figure 3A). The detected very small carbon peak is probably due to the adsorbed ambient CO_2_ on the surface (Samadi et al., 2014). As shown in Figure 3B, two peaks at 1021.2 eV and 1044.3 eV attribute to the conveyed binding energies of Zn 2p_3/2_ and Zn 2p_1/2_ that indicates the presence of Zn^2+^ state (Naseri et al., 2017). Besides, these two peaks' difference of 23.1 eV also confirmed that ZnO was generated (Qiao et al., 2016). Figure 3C shows that the O 1 s peak located at 530.1 eV was assigned to the lattice oxygen O^2−^ and that the other peak at 531.8 eV was ascribed to the adsorbed hydroxyl groups (Al-Gaashani et al., 2013; Yang et al., 2013). The BET surface area and pore size distribution of the porous ZnO were analyzed by nitrogen adsorption-desorption. Supplementary Figure 3A shows that the porous structure is highly possibly from the space between particles. The BET specific surface area is determined to be up to 31.4 m^2^/g. Correspondingly, Supplementary Figure 3B shows that the pore size distributions calculated by the BJH method displays a mean size of ~18 nm.

### Effect of Calcination Temperatures and BPA Concentrations

**Figure 4:**
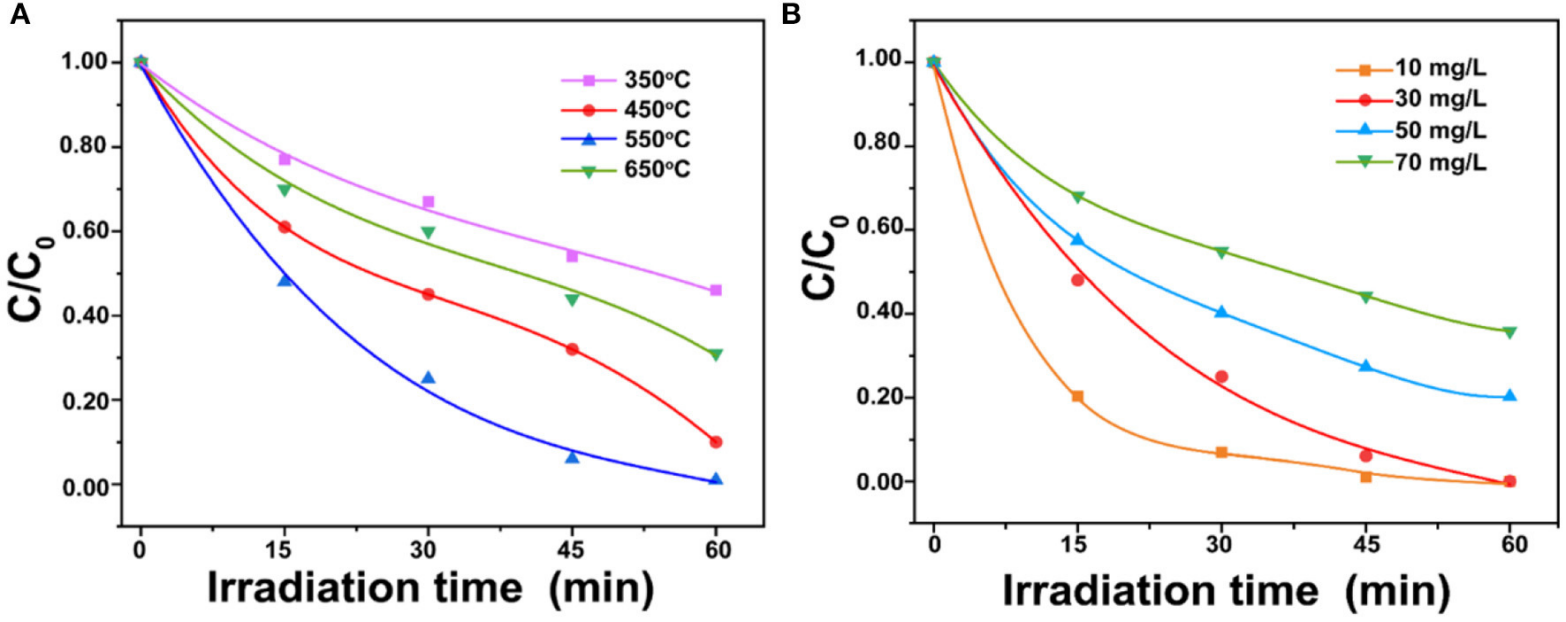
**(A)** Effect of porous ZnO photocatalysts synthesized under different calcination temperatures; **(B)** Effect of BPA concentrations on photodegradation (ZnO dosage = 1.0 g/L and pH = 6.5).

As shown in Figure 4A, with the calcined temperature of ZnO increased from 350° to 550°C, the degradation efficiency of BPA increased. The maximum degradation efficiency (99%) in 1 h was reached at 550°C. The superior performance is possibly related to the higher crystallinity than the ZnO calcined at 350°C and the greater number of pore structures than the ZnO calcined at 450° and 650°C. The high crystallinity is a benefit for photoactivity and the pore structure is a benefit for the adsorption and photodegradation of BPA. The comparison between ZnO photocatalysts for BPA degradation prepared in this study with those reported in the literature was listed in Supplementary Table 1. It showed that the porous ZnO photocatalyst synthesized in this study possessed the best performance. Figure 4B shows degradation efficiencies of different initial BPA concentrations from 10 to 70 mg/L. The result showed that ~99% BPA can be removed in 1 h at the concentration of 30 mg/L. However, the decline of the degradation efficiency is observed with the increased BPA concentration. This is possibly due to the fact that the active catalytic sites supplied by 50 mg porous ZnO are not enough for the degradation of a higher BPA concentration. Besides, high BPA concentration can adsorb light energy and inhibit photons to activate the porous ZnO photocatalyst (Ani et al., 2018).

### Effect of ZnO Dosages

**Figure 5:**
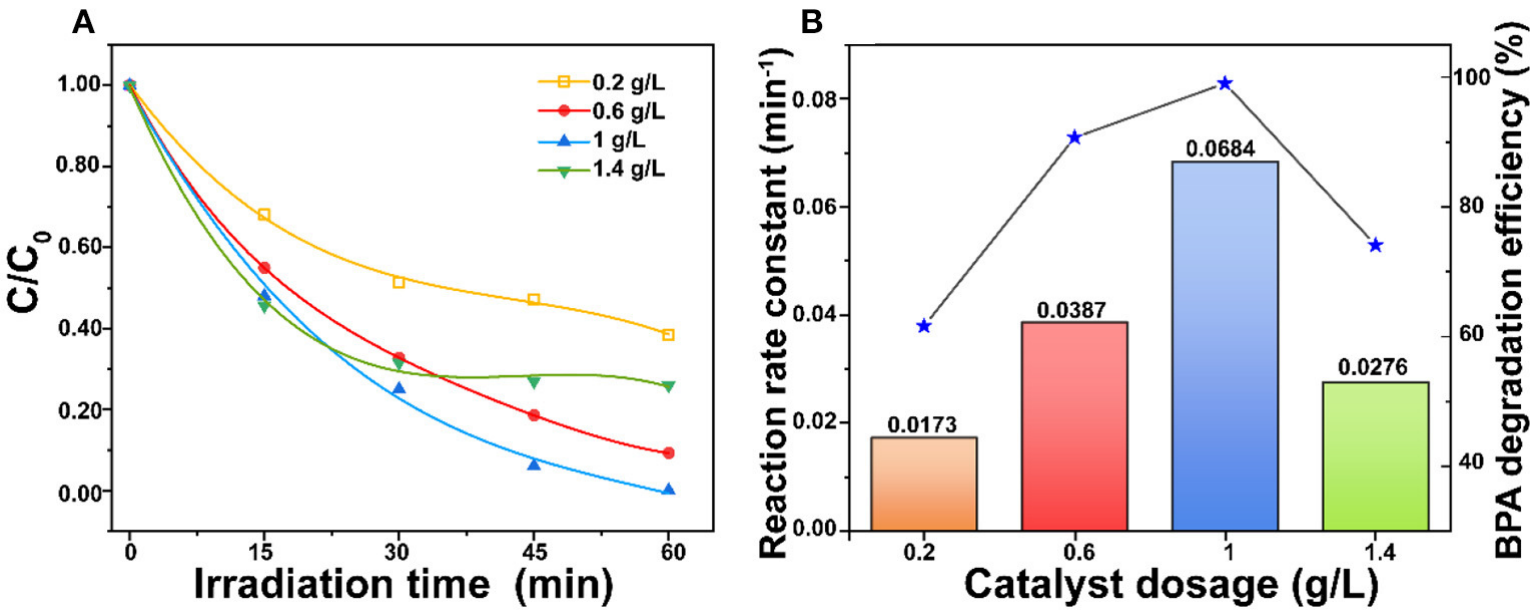
**(A)** Comparison of ZnO dosages and **(B)** Effect of ZnO dosages on reaction rate constant for photodegradation of BPA under simulated sunlight irradiation (BPA concentration = 30 mg/L and pH = 6.5).

Figure 5 shows the influence of ZnO dosages on the BPA photodegradation. The result shows that the reaction rate constant increased from 0.0173 to 0.0684 min^−1^ with the ZnO dosage increased from 0.2 to 1.0 g/L. However, the reaction rate constant was then decreased to 0.0276 min^−1^ when the ZnO dosage rose to 1.4 g/L. It is well-known that increasing the ZnO dosage can generate more radicals for highly efficient BPA photodegradation (Ghasemi et al., 2016). However, an excess amount of the ZnO photocatalyst could lead to negative effects. The high suspension might inhibit the penetration of photons and enhance the tendency of agglomeration, which would reduce the effective surface area of ZnO for light absorption (Ling et al., 2015). The trade-off between these two opposing effects led to the optimum catalyst dosage at 1.0 g/L in this study. Moreover, 50 μl and 100 μl 30 wt.% H_2_O_2_ were added to hinder the recombination of photo-generated electrons and holes to improve the BPA photodegradation. However, Supplementary Figure 4 shows that H_2_O_2_ hinders the photocatalytic degradation of BPA. The possible reasons are that (1) H_2_O_2_ consumed OH^−^ and h^+^ which were reactive species, (2) there was adsorption competition between H_2_O_2_ and BPA on the porous ZnO, and (3) H_2_O_2_ was adsorbed onto ZnO surface to result in adverse modification (Dougna et al., 2015).

### Effect of Original pH Value

**Figure 6:**
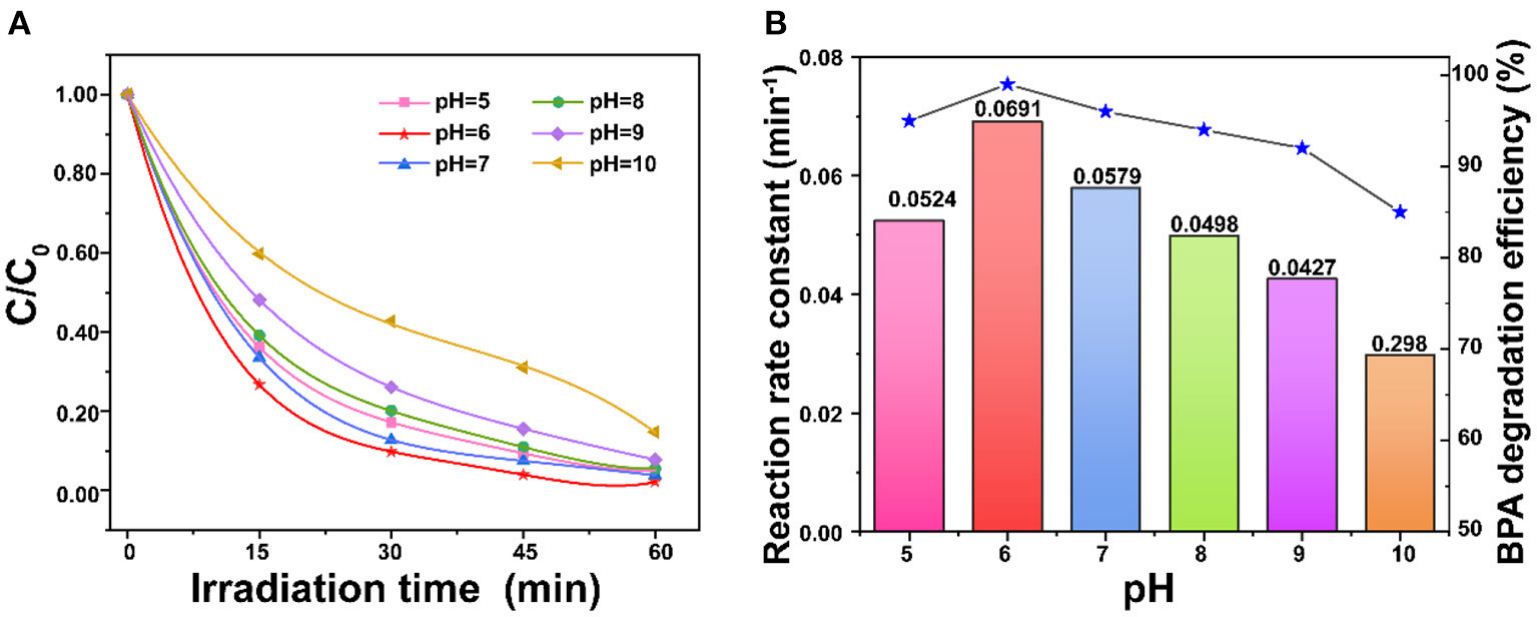
**(A)** Comparison of pH on degradation of BPA; **(B)** Effect of pH on the reaction rate constant over porous ZnO under simulated sunlight irradiation (BPA concentration = 30 mg/L and ZnO dosage = 1 g/L).

In this study, the sample pH was adjusted by HCl and NaOH to analyze the effect of different pH values on the degradation efficiency. The results show that pH of 10.0 led to the minimum degradation efficiency (85%), and the degradation efficiency increased with the pH decrease to 6.0 (Figure 6A). The maximum degradation efficiency reached at pH of 6.0. This is possibly due to the fact that the photodegradation performance of BPA is affected by ions in the solution and also the charges on ZnO photocatalyst and BPA. When pH <6.0, the anionic Cl^−^ could compete with the adsorption of BPA, which reduces the degradation efficiency. On the other hand, ions generated from NaOH also competed with the adsorption of BPA (Sin et al., 2013). In addition, the zero-point charge of ZnO is reported at around pH 9.0 (Xu et al., 2020). The pKa values of BPA are 9.6 and 10.2 (Nguyen et al., 2019). The reactions on the ZnO surface at different pH were shown in Figure 6B and SI. When pH >9.0, the porous ZnO becomes deprotonated to result in the repulsion between the ZnO and anionic BPA for the low degradation at high pH.

### Effect of Inorganic Ions

**Figure 7:**
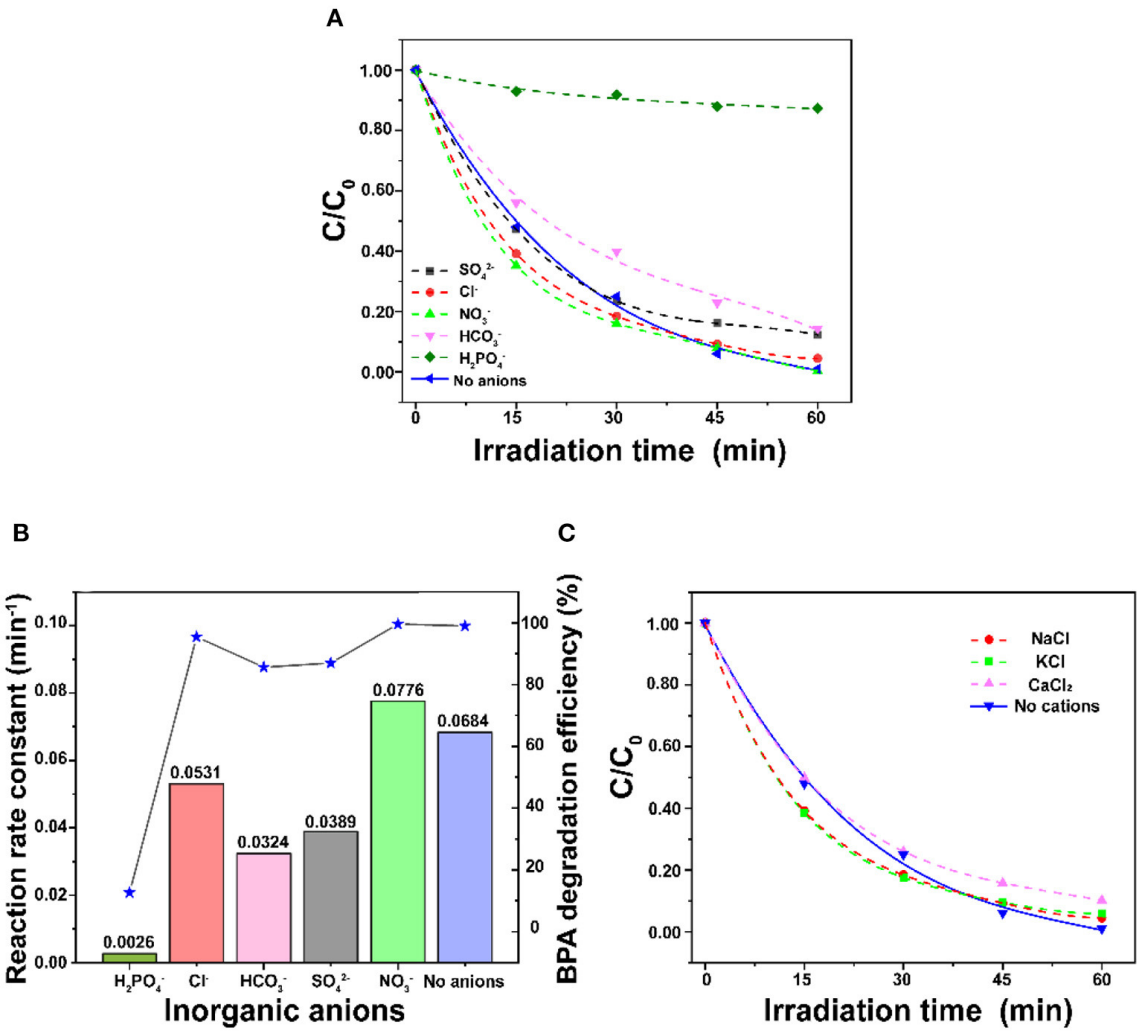
**(A)** Comparison of different inorganic anions on the degradation of BPA, **(B)** Effect of inorganic anions on the reaction rate constant, and **(C)** Comparison of different inorganic cations on the degradation of BPA (BPA concentration = 30 mg/L and ZnO dosage = 1 g/L).

Figure 7 shows the degradation performance in the presence of inorganic ions. Figure 7A shows that the BPA degradation process is inhibited at different levels after the addition of 10 mM H_2_${\text{PO}}_{4}^{-}$, ${\text{HCO}}_{3}^{-}$, ${\text{SO}}_{4}^{2-}$, and Cl^−^ anions. Especially, H_2_${\text{PO}}_{4}^{-}$ remarkably inhibited the degradation of BPA to lead to only 10% degradation efficiency; the rate constant was decreased from 0.0684 to 0.0026 min^−1^. Cl^−^ exhibits the minimal inhibition which decrease the rate constant to 0.0531 min^−1^ (Figure 7B). Similar to the trend found by Tang et al. (2018), the inhibition effects for the photocatalytic degradation of BPA found in this work were decreased in the order H_2_${\text{PO}}_{4}^{-}$ > ${\text{HCO}}_{3}^{-}$ > ${\text{SO}}_{4}^{2-}$ > Cl^−^. The main reason is that anions are generally considered to be scavengers of hydroxyl radicals and photo-holes. However, it is noteworthy that the reaction rate increased to 0.0776 min^−1^ with the addition of 10 mM ${\text{NO}}_{3}^{-}$. The possible reason is that nitrate is the primary precursor of hydroxyl radicals, which are the strong reactive species for the degradation of BPA (Gao et al., 2017). As shown in Figure 7C, the degradation efficiency of BPA is just slightly decreased with the addition of 10 mM of K^+^, Ca^2+^, and Na^+^. It was reported that K^+^, Ca^2+^, and Na^+^ could reduce the thickness of electrical double layer to suppress electrostatic repulsion between porous ZnO to enhance the aggregation (Zhao et al., 2018). On the other hand, the adsorbed cations could generate a screening effect that benefits from dispersion interactions between BPA and the ZnO photocatalyst (Liu et al., 2016). Thus, the trade-off between the porous ZnO aggregation and the positive effect caused by screening effect might contribute to a very slight change to the degradation of BPA.

### Stability of the ZnO Photocatalyst

**Figure 8:**
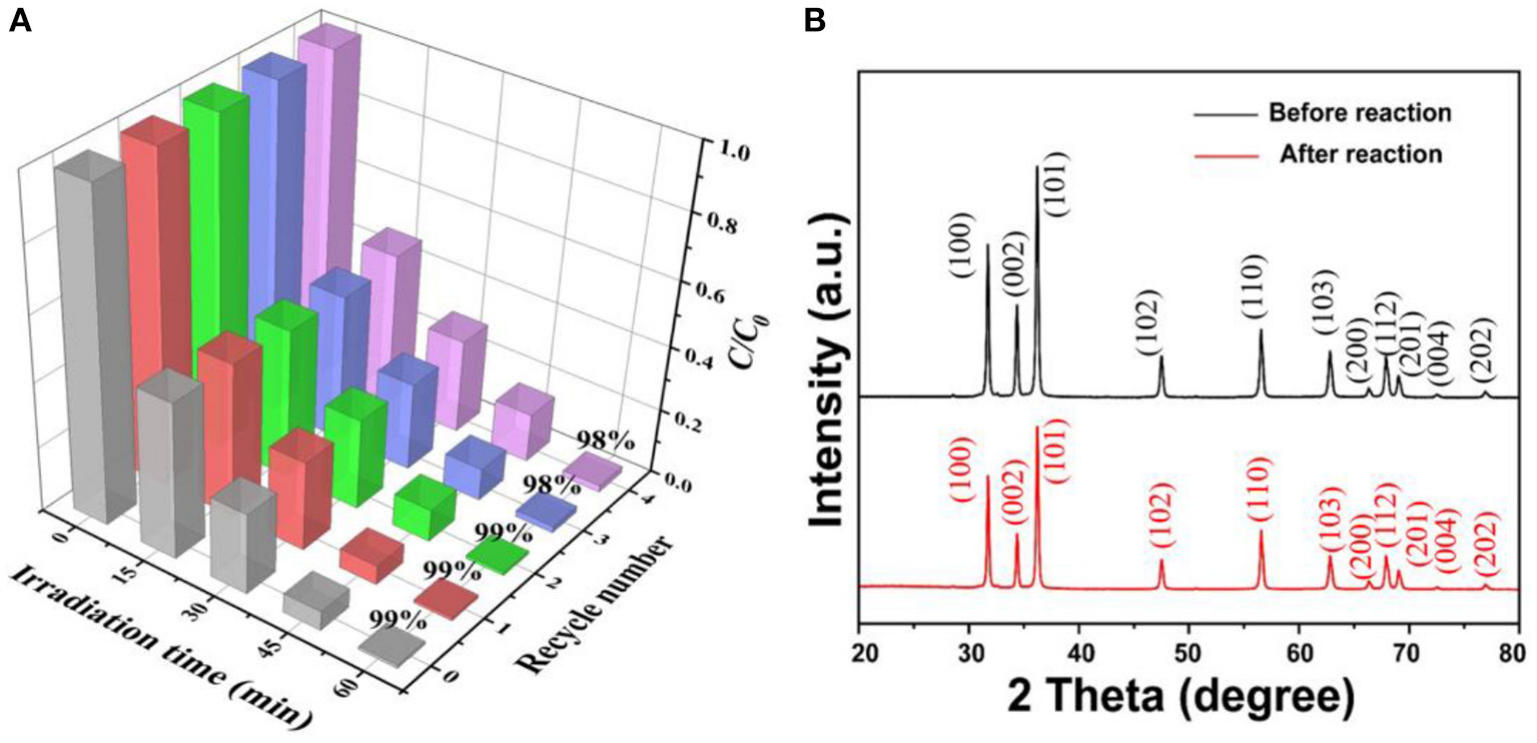
**(A)** Photocatalytic degradation of BPA over the ZnO photocatalyst over five cycles; **(B)** XRD patterns of the ZnO photocatalyst after five cycles.

The stability of the ZnO photocatalyst is an important criterion for practical applications. In this study, five cycles of photodegrading BPA were conducted under the same condition to investigate the stability of the porous ZnO photocatalyst. After each cycle of photocatalytic degradation of BPA in 1 h, the porous ZnO was washed with absolute ethanol and ultrapure water, and then centrifuged and dried. As shown in Figure 8A, there is only a very slight decline for the BPA degradation efficiency after three cycles confirming that the porous ZnO photocatalyst is stable. Moreover, the XRD spectra and SEM of the porous ZnO photocatalyst after five cycles were also compared. Figure 8B and Supplementary Figure 5 show that no obvious difference in the diffraction peak, structure, and morphology is observed, thus confirming the stability of the ZnO photocatalyst.

### Roles of Reactive Species

**Figure 9:**
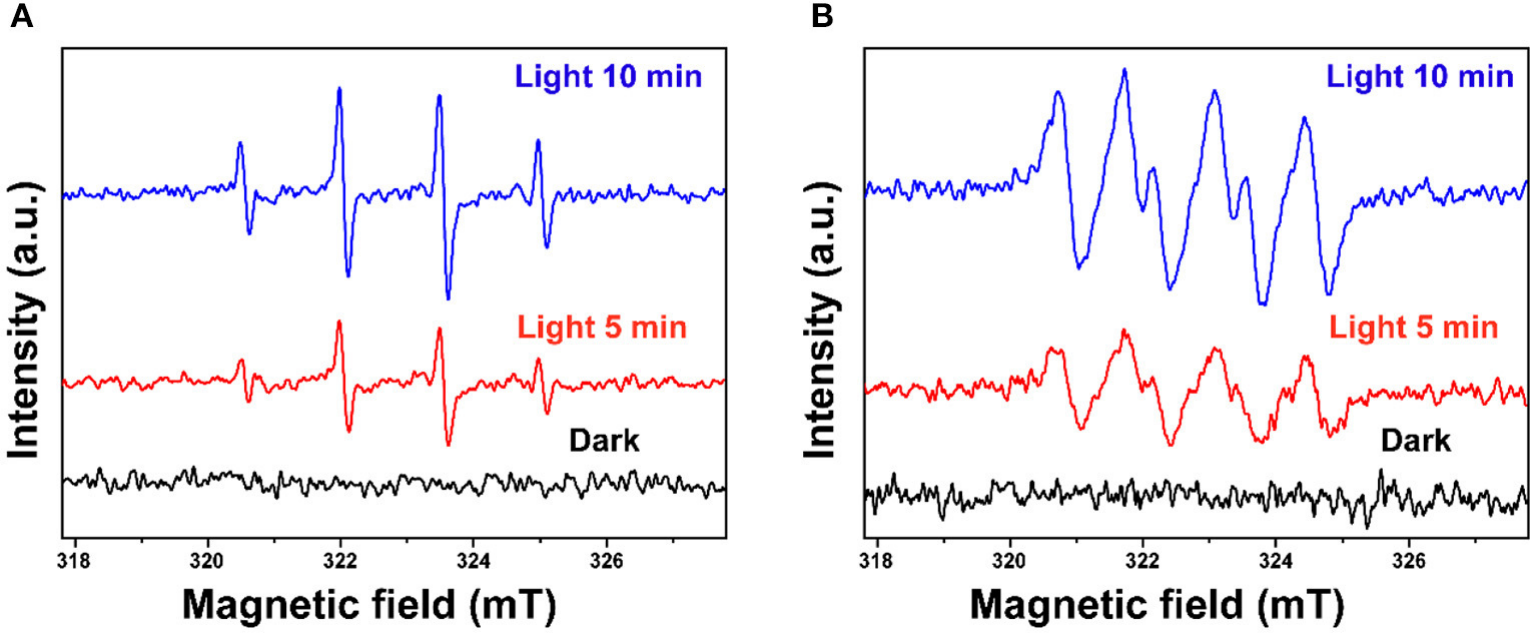
ESR spectra of **(A)** DMPO-·OH and **(B)** DMPO-·${\text{O}}_{2}^{-}$ under light and in the dark.

In this study, scavenging experiments were conducted to investigate reactive species formed in the photocatalytic degradation process. Ammonium oxalate (AO), t-butyl alcohol (TBA), p-benzoquinone (BQ), and dimethyl sulfoxide (DMSO) were added to scavenge h^+^, ·OH, ·${\text{O}}_{2}^{-}$ and e^−^, respectively. As shown in Supplementary Figure 6, the photocatalytic degradation of BPA is inhibited differently by various scavengers which illustrates that different reactive species play different roles. It was inhibited substantially by AO, BQ, and DMSO revealing that h^+^, ·${\text{O}}_{2}^{-}$, and e^−^ play the key role for BPA degradation. Besides, ·OH also contributed in a certain way to the degradation of BPA. The existence of ·OH and ·${\text{O}}_{2}^{-}$ was further confirmed using the ESR technique with DMPO as the spin trap (Wang et al., 2020a). As shown in Figure 9, the characteristic peaks of DMPO-·OH and DMPO-·${\text{O}}_{2}^{-}$ are obviously detected under simulated sunlight irradiation, while no signal is found in the dark. These results revealed that ·OH and ·${\text{O}}_{2}^{-}$ were formed for the photocatalytic degradation of BPA over the as-fabricated ZnO photocatalyst.

### Possible BPA Degradation Pathways

**Figure 10:**
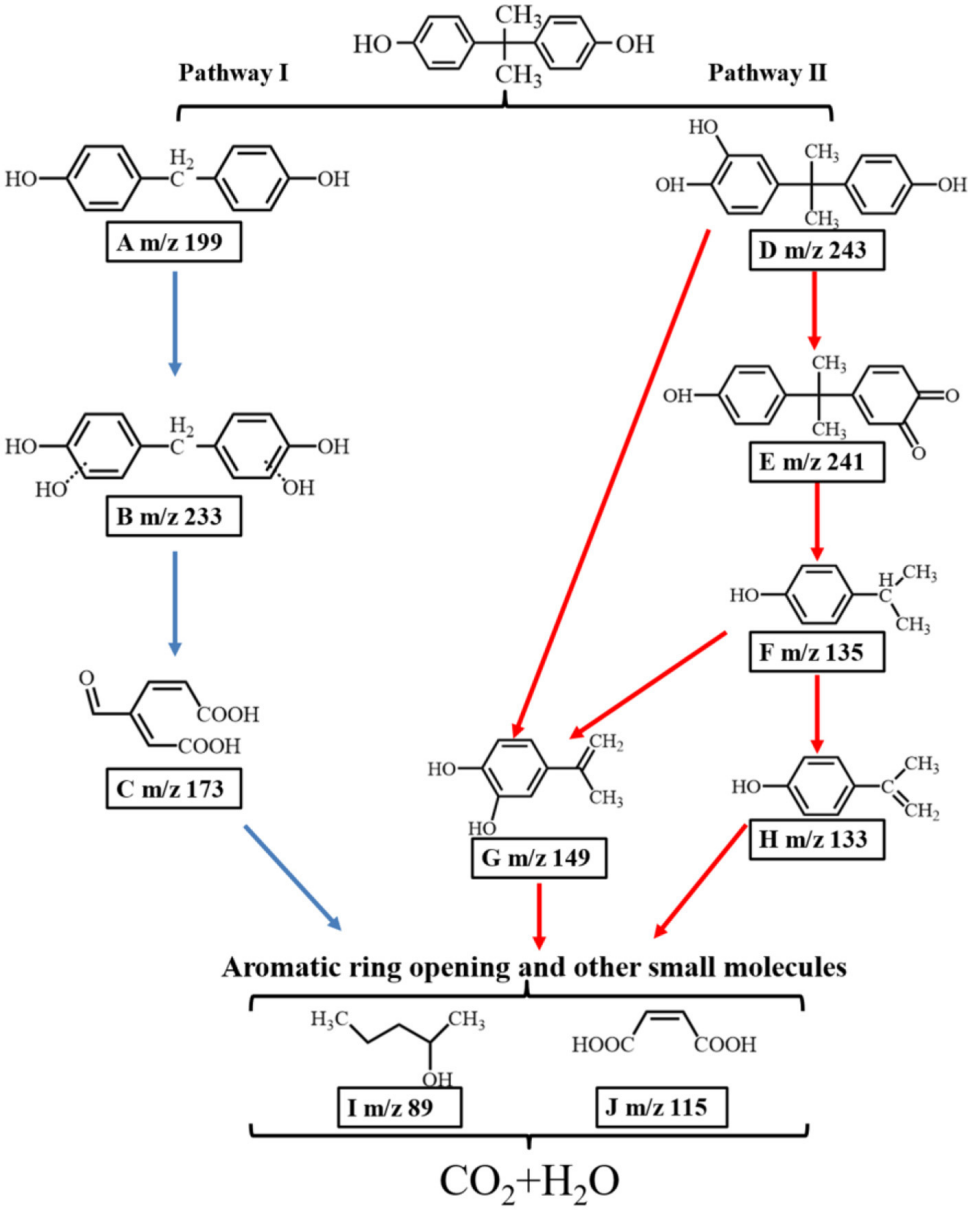
Two proposed photocatalytic degradation pathways of BPA over the ZnO photocatalyst.

As shown in Supplementary Figure 7 and Supplementary Table 2, several reaction intermediates were detected in this study. Based on present LC-MS results and the previous literature, two possible pathways of the degradation of BPA over the porous ZnO are proposed in Figure 10. In pathway I, radicals in the aqueous solution attacked the electron-rich C-C bound to generate intermediate A (m/z 199) (Xu et al., 2018). The attack of electrophilic ·OH formed intermediate B (m/z 233). Intermediate C (m/z 173) was produced by the route of BPA → A → B → C via oxidation reaction for the cleavage of the C-C bridge and the aromatic ring. In pathway II, the electrophilic ·OH group attacked the aromatic ring of the BPA, resulting in the formation of hydroxylated intermediate D (m/z 243). Secondly, intermediate E (m/z 241) was formed through the hydroxylation and dehydration (Li et al., 2016). It was reported that reactive species could attack the C-C bond between the two aromatic rings to form intermediate F (m/z 135) (Zhu et al., 2018). At the same time, intermediate G (m/z 149) was produced by the route of BPA → D → E → F → G or BPA → D → G (Du et al., 2016). Intermediate H (m/z 133) was produced by the dehydrogenation process of F (m/z 135) (Diao et al., 2018). Finally, ring opening products including I (m/z 89) and J (m/z 115) were formed, which were further mineralized into CO_2_ and H_2_O. Besides, the total organic carbon (TOC) test was carried out to investigate the mineralization rate of BPA over the porous ZnO. As shown in Supplementary Figure 8, the mineralization rate of BPA after 60 min is 54.7%.

## Conclusion

In this study, we have successfully developed a porous ZnO photocatalyst with core-shell structure and a large surface area for efficient removal of BPA. Photocatalytic performance was confirmed to be closely related to calcination temperatures, BPA concentrations, ZnO dosages, and pH. In particular, the porous core-shell ZnO calcined under 550°C exhibited the maximum catalytic activity, obtaining 99% degradation of BPA in 1 h under the conditions of 30 mg/L BPA, 1 g/L ZnO and pH of 6.5. Furthermore, reasonable degradation pathways of BPA were proposed based on the determined intermediates, mainly including the C-C bridge cleavage, aromatic ring cleavage, hydroxylation, dehydrogenation, etc. The superior catalytic activity of the as-prepared ZnO photocatalyst mainly benefited from the porous core-shell structure with a large surface area, which was able to improve mass-transfer efficiency, promote light absorption, and expose more active sites. This study demonstrates that the prepared porous ZnO photocatalyst has high photoactivity and stability and is a promising photocatalyst for the degradation of endocrine disrupting compounds.

## Data Availability Statement

The original contributions presented in the study are included in the article/Supplementary Materials, further inquiries can be directed to the corresponding author/s.

## Author Contributions

All authors participate this work and all authors have agreed to publish this work.

## Conflict of Interest

The authors declare that the research was conducted in the absence of any commercial or financial relationships that could be construed as a potential conflict of interest.
