# Supplementary material for: Facile Synthesis of Porous ZnO Nanoparticles Efficient for Photocatalytic Degradation of Biomass-Derived Bisphenol A Under Simulated Sunlight Irradiation
Source: Front Bioeng Biotechnol. 2021 Jan 14;8:616780. doi: 10.3389/fbioe.2020.616780 (PMC7841389; doi:10.3389/fbioe.2020.616780)
Supplement: Supplementary file 1 [file Data_Sheet_1.docx]

Supplementary Material

Facile synthesis of porous ZnO nanoparticles efficient for photocatalytic degradation of biomass-derived bisphenol A under the simulated sunlight irradiation

Yujie Wang^1,†^, Kang Hu^2,3,†^, Zhiyu Yang^2^, Chenlu Ye^2^, Xin Li^2^, Ruiqi Li^2^, Kai Yan^2,*^

^1^Guangzhou Key Laboratory of Environmental Catalysis and Pollution Control, School of Environmental Science and Engineering, Institute of Environmental Health and Pollution Control, Guangdong University of Technology, Guangzhou 510006, China

^2^Guangdong Provincial Key Laboratory of Environmental Pollution Control and Remediation Technology, School of Environmental Science and Engineering, Sun Yat-sen University, Guangzhou 510006, P.R. China

^3^Ganjiang Innovation Academy, Chinese Academy of Sciences, Ganzhou 341100, P.R. China

*** Correspondence:**Kai Yan
[yank9@mail.sysu.edu.cn](mailto:yank9@mail.sysu.edu.cn)

**†**These authors contribute equally to this work

**Text S1: Materials characterization**

The crystal structure characteristics of porous ZnO photocatalyst were analyzed via the X-ray diffractometer (XRD, Rigaku, UltimaIV) with Cu Kα irradiation (k = 0.15406 nm). The data was obtained with a step width of 0.02°/2θ from 5° to 90°. X-ray photoelectron spectroscopy (Thermo Fisher Scientific, ESCALab250) with Al Kα source was used to analyze the chemical states. The charging shift was corrected using C 1s binding energy of hydrocarbon (284.8 eV) as the standard. Scanning electron microscopy (SEM) images were got from a ZEISS GeminiSEM 500 system. A transmission electron microscope (FEI, Tecnai G2 F30) was applied at accelerating voltages of 200 kV to analyze the microscopic morphology. Further morphology analysis was performed by high resolution transmission electron microscope (FEI, Titan G2 60-300). Electron spin resonance spectra (ESR) was acquired on a JES-X320 (JEOL, Japan).

**Text S2: The calcination process of porous ZnO**

The calcination of porous ZnO may proceed along the following reactions. Firstly, the urea began to hydrolyze to give off ammonia, and formed OH^-^ anions and CO_2_ according to the Eqs. (1) and (2). The OH^-^ and CO_2_ further reacted with Zn^2+^ ions which were released from zinc acetate dihydrate in the solution, and generated zinc carbonate hydroxide according to the Eq. (3). Moreover, the Zn^2+^ cations were converted into crystal nuclei, then the sheet-like architecture was formed during the reaction time process (Miao et al., 2016). Then, the porous and core-shell structure was obtained by calcining the precursor.

CO(NH_2_)_2_ + 3H_2_O → 2NH_3_·H_2_O + CO_2_ (1)

2NH_3_·H_2_O → NH_4_^+^ + OH^-^ (2)

5Zn^2+^ + 10OH^-^ + 2CO_2_ + 2H_2_O → Zn_5_(CO_3_)_2_(OH)_6_ + 4H_2_O (3)

Zn_5_(CO_3_)_2_(OH)_6_·→ 5ZnO +2CO_2_ + 3H_2_O (4)

**Text S3: ZnO surface reaction in different pH**

When pH is lower than the ZnO isoelectric point of 9.0, the surface is protonated according to Eq. (5). When at higher pH, it is deprotonated according to the Eq. (6).

ZnOH+H^+^ → ZnOH_2_^+^ (5)

ZnOH+OH^-^ → ZnO^-^+H_2_O (6)


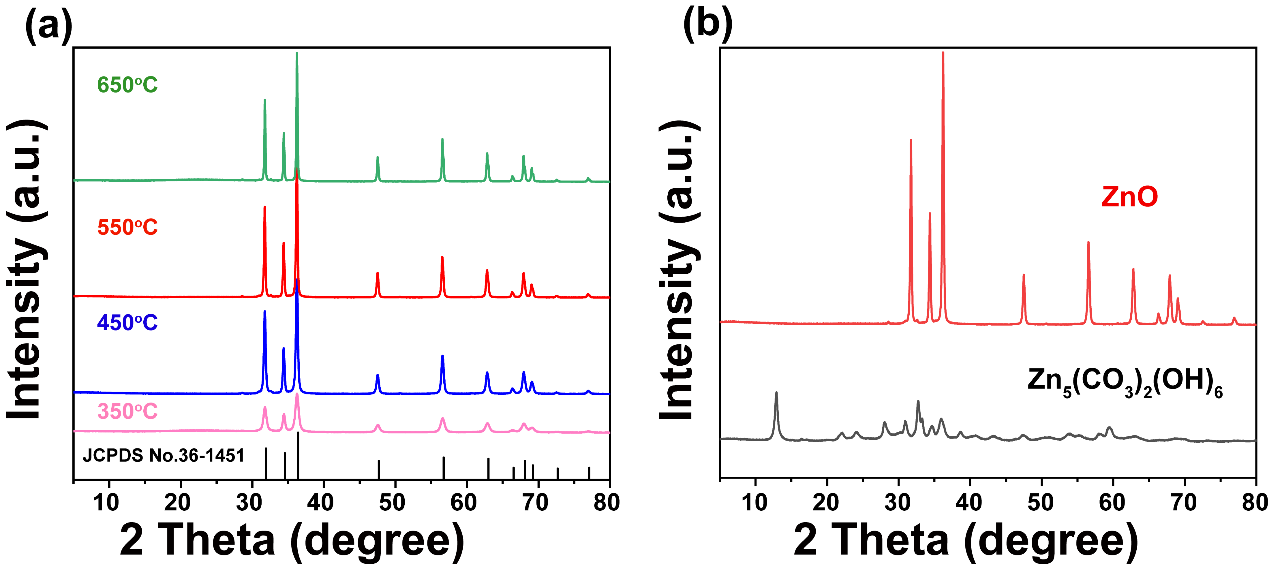


Fig. S1. XRD patterns of (a) porous ZnO prepared under different calcination temperatures; (b) The precursor before and after calcination under 550^o^C.


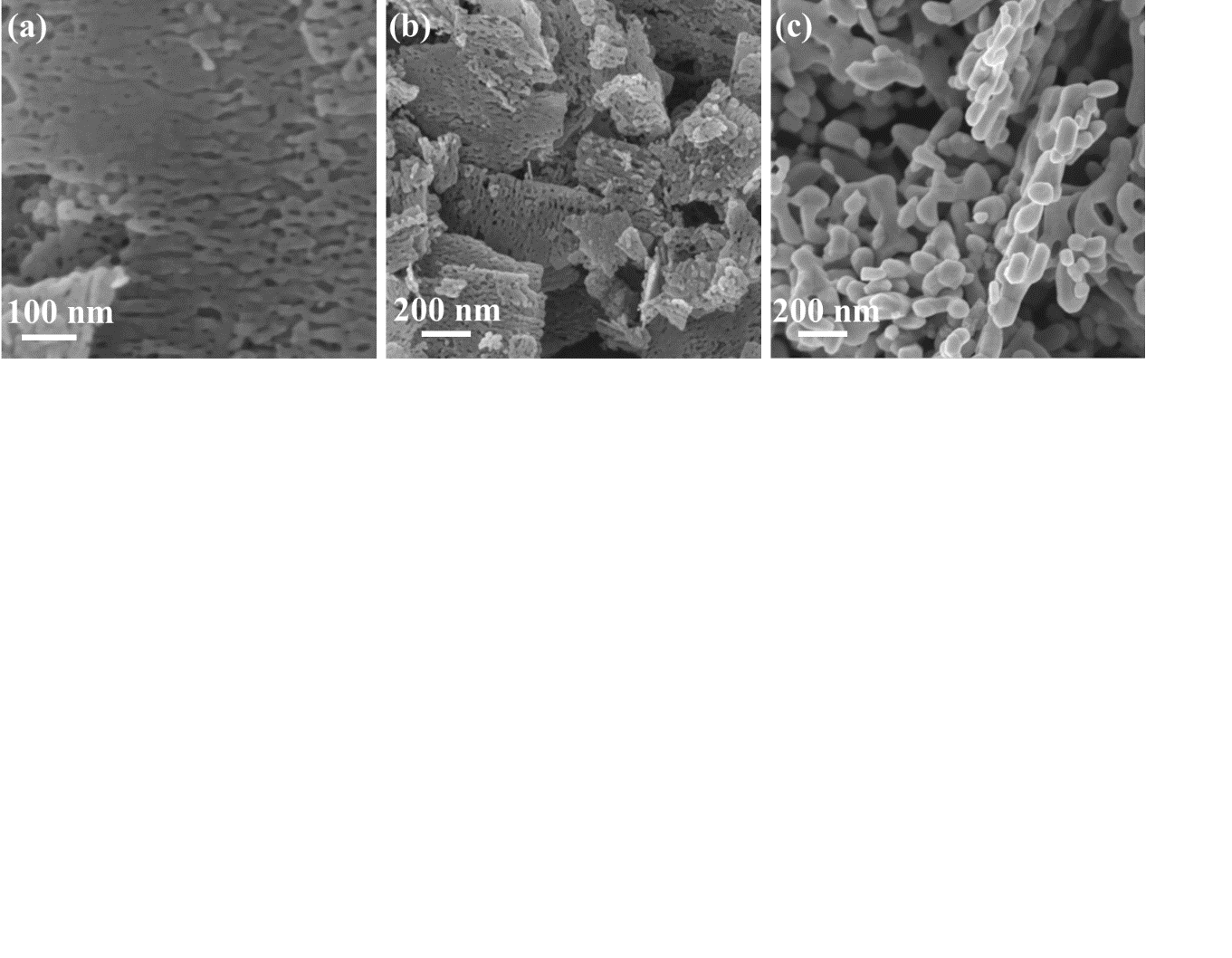


Fig. S2. SEM images of the as-prepared ZnO samples under different calcination temperatures: (a) 350^o^C, (b)450^o^C and (c) 650^o^C.


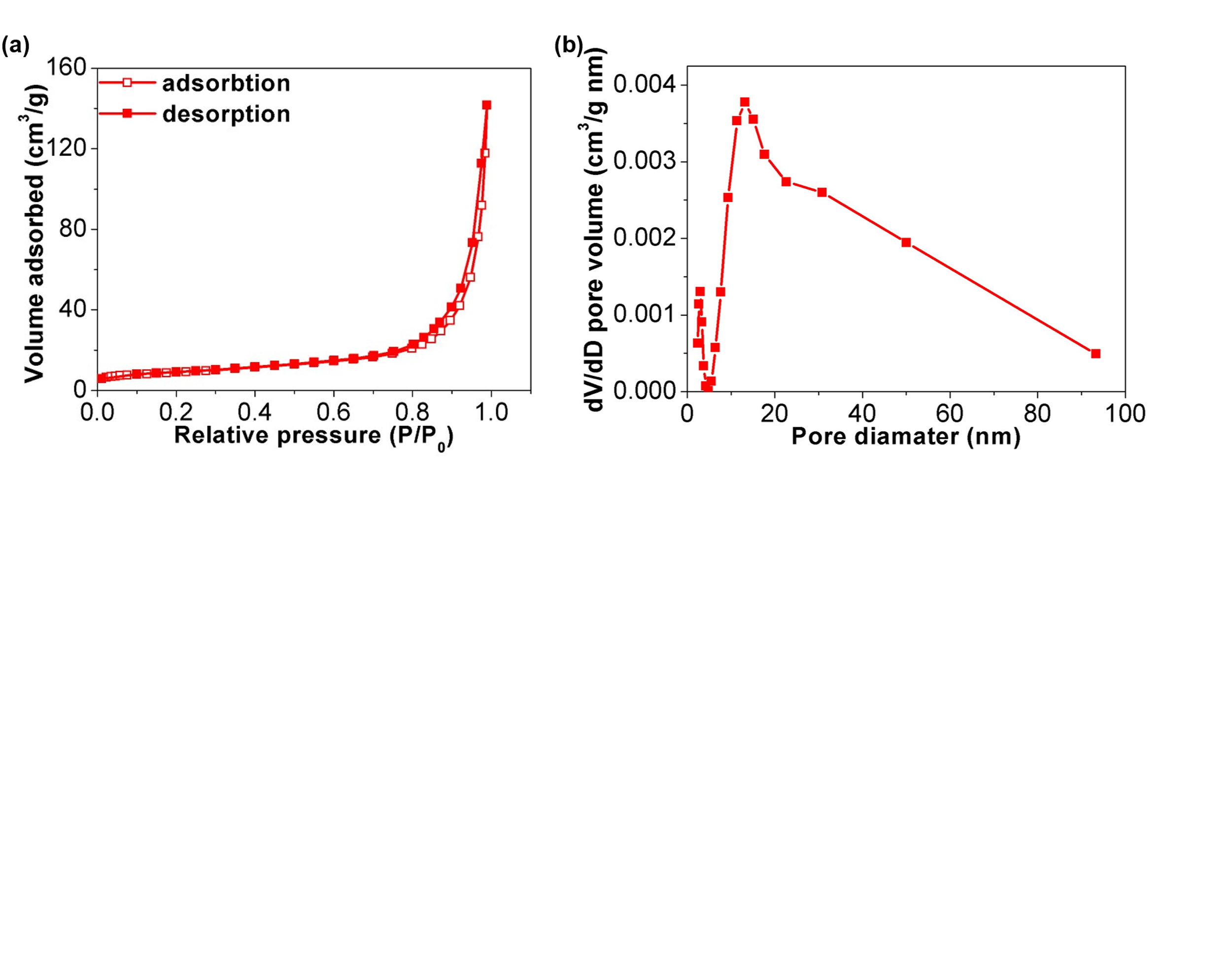


Fig. S3. (a) Nitrogen adsorption and desorption isotherm curve and (b) pore size distribution of ZnO.


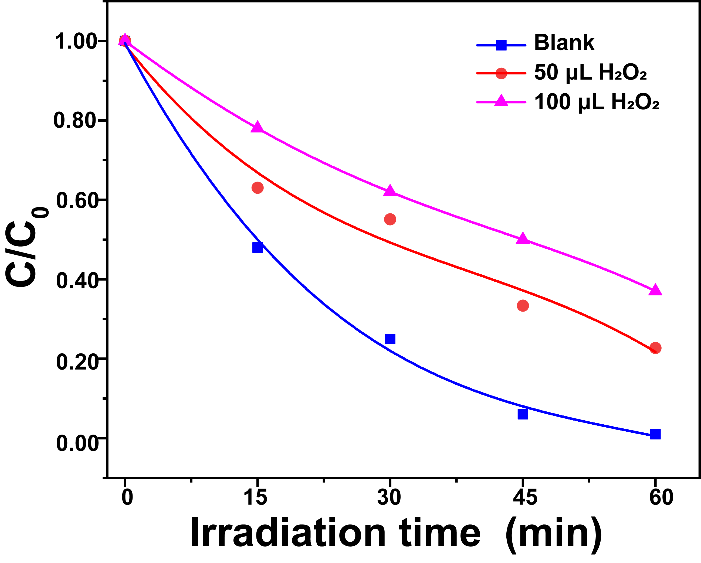


Fig. S4. Effect of H_2_O_2_ content on the degradation of BPA over porous ZnO.


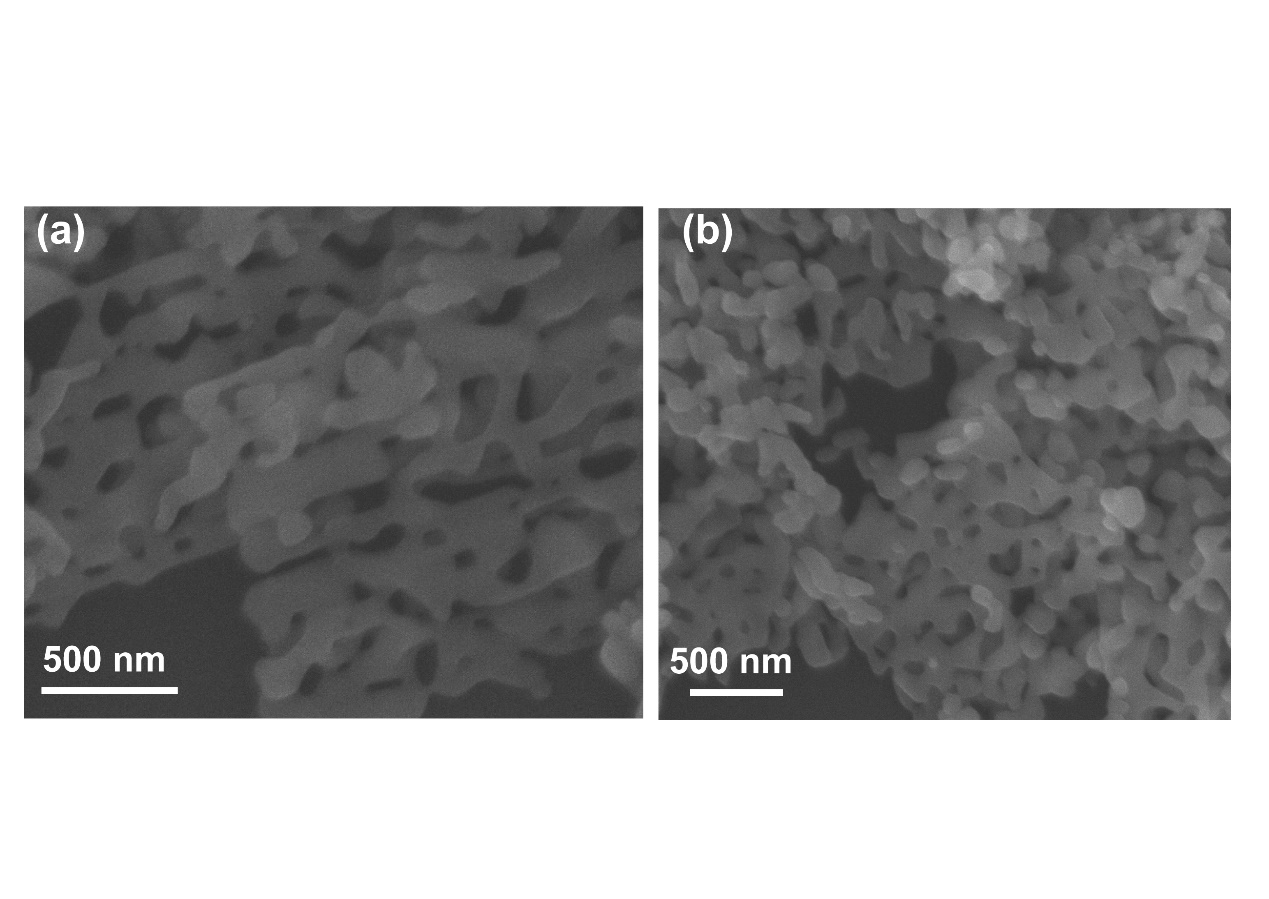


Fig. S5. SEM images of ZnO: (a) recycle once and (b) recycle 5 times.


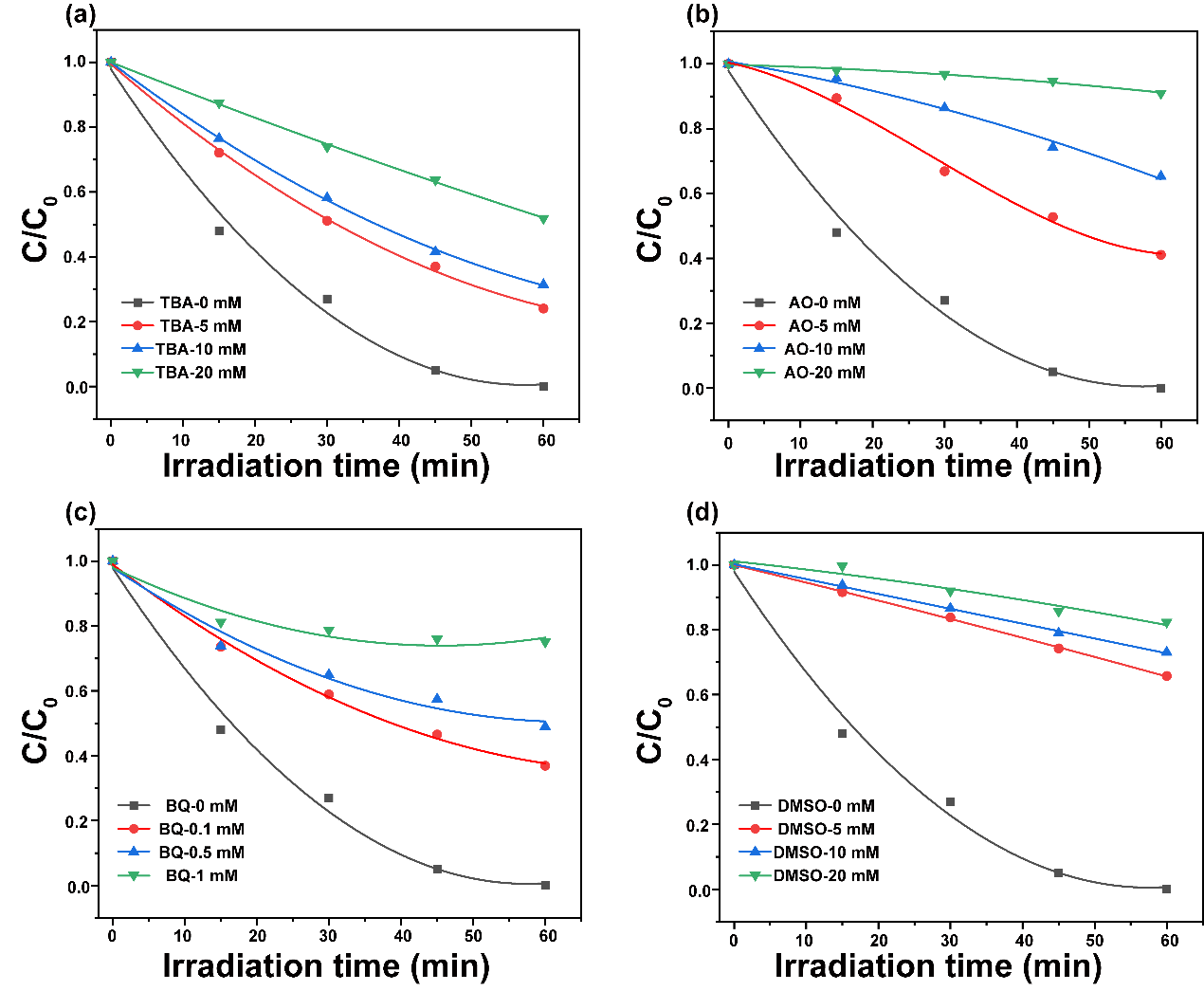


Fig. S6. Effect of different concentrations of (a) TBA (b) AO (c) BQ (d)DMSO on the degradation of BPA over the porous ZnO under the simulated sunlight irradiation.


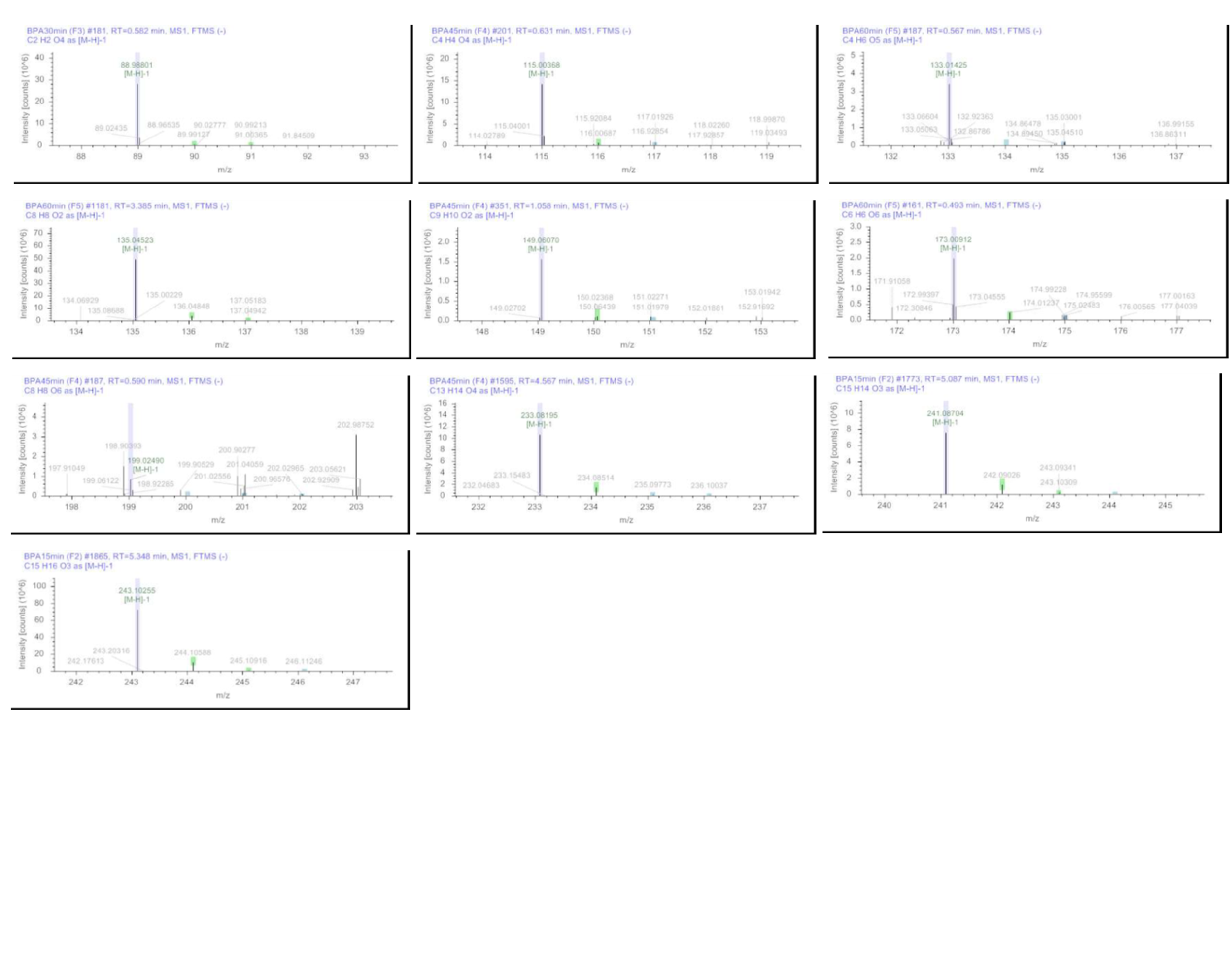
Fig. S7. Mass spectra of degraded intermediates of BPA.


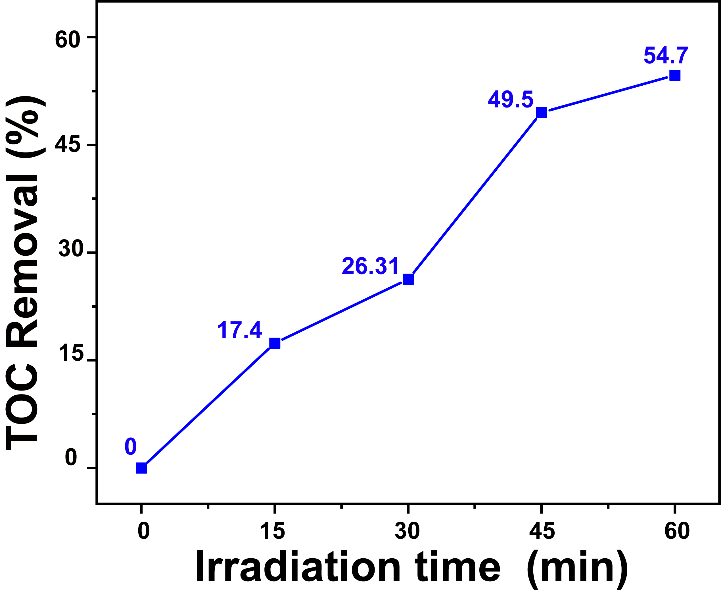


Fig. S8. The TOC change during the photodegradation of BPA over porous ZnO.

**Table S1.** The comparison between ZnO-based photocatalysts for BPA degradation prepared in this study with partial updated reported literatures.

| **Materials** | **Light source** | **Degradation efficiency** | **Catalyst**  **dosage (g/L)** | **BPA Concentration**  **(mg/L)** | **Ref.** |
| --- | --- | --- | --- | --- | --- |
| 1% Ag-doped ZnO (molar ratio) | UVlamp  (Apelex, VL-15C, λ=254 nm, 30 W) | 100% in 25 h | 1.0 | 50 | (Bechambi et al., 2015a) |
| 2% Ce-doped ZnO (molar ratio) | UVlamp  (Apelex, VL-15C, λ=254 nm, 30 W) | 100% in 24 h | 1.0 | 50 | (Bechambi et al., 2016; Bechambi et al., 2015c) |
| 4% C-doped ZnO  (molar ratio) | UVlamp  (Apelex, VL-15C, λ=254 nm, 30 W) | 100% in 24 h | 1.0 | 50 | (Bechambi et al., 2015b) |
| ZnO (Dae Jung Co., Korea) | UV light (254 nm, 120 W) | 92% in 4 h | 0.05 | 100 | (An et al., 2018) |
| Ce-doped ZnO | sunlight | 97.8% in 8 h | 0.8 | 10 | (Kamaraj et al., 2014) |
| La-doped ZnO | mercury lamps (8 × 8 W) | 100% in 4 h | 0.3 | 200 | (Selvam et al., 2013) |
| Coupled zinc-iron oxide | UV-A lamp (8 W) | 99% in 210 min | 0.5 | 50 | (Tan et al., 2015) |
| 1% w/w Ag/ZnO | UV lamp (3UVTM lamp, UVP, λ=302 nm) | 86% in 2 h | 1.0 | 10 | (Jasso-Salcedo et al., 2014) |
| N-doped ZnO | Xenon arc lamp (350 W) | 93% in 4 h | 0.3 | 20 | (Qiu et al., 2013) |
| ZnO doped with zinc-hexacyanoferrate | sunlight | 97% in 24 h | 1.0 | 2 | (Rani and Shanker, 2018) |
| ZnO | sunlight | 75% in 24 h | 1.0 | 2 | (Rani and Shanker, 2018) |
| Iron hexacyanoferrate encapsulated with zinc oxide (ZnO@FeHCF) | sunlight | 94% in 24 h | 1.6 | 2 | (Rachna et al., 2019) |
| **Porous ZnO** | **Xenon lamp (300W)** | **99% in 1 h** | **1.0** | **30** | **This study** |

**Table S2.** The intermediates detected in the photocatalytic degradation of BPA.

| Intermediates | Molecular weight (m/z) | structure | Reported by |
| --- | --- | --- | --- |
| BPA | 228 |  |  |
| A | 199 |  | (Xu et al., 2018) |
| B | 233 |  | (Li et al., 2015) |
| C | 173 |  | (Gao et al., 2017) |
| D | 243 |  | (Li et al., 2016) |
| E | 241 |  | (Li et al., 2016) |
| F | 135 |  | (Zhu et al., 2018) |
| G | 149 |  | (Diao et al., 2018) |
| H | 133 |  | (Zhang et al., 2014) |
| I | 89 |  | (Peng et al., 2017) |
| J | 115 |  | (Sharma et al., 2015) |

**References**

An, S.-N., Choi, N.-C., Choi, J.-W., Lee, S., 2018. Photodegradation of Bisphenol A with ZnO and TiO_2_: Influence of metal ions and fenton process. Water Air Soil Poll. 229, 43.

Bechambi, O., Chalbi, M., Najjar, W., Sayadi, S., 2015a. Photocatalytic activity of ZnO doped with Ag on the degradation of endocrine disrupting under UV irradiation and the investigation of its antibacterial activity. Appl. Surf. Sci. 347, 414-420.

Bechambi, O., Jlaiel, L., Najjar, W., Sayadi, S., 2016. Photocatalytic degradation of bisphenol A in the presence of Ce-ZnO: Evolution of kinetics, toxicity and photodegradation mechanism. Mater. Chem. Phys. 173, 95-105.

Bechambi, O., Sayadi, S., Najjar, W., 2015b. Photocatalytic degradation of bisphenol A in the presence of C-doped ZnO: Effect of operational parameters and photodegradation mechanism. J. Ind. Eng. Chem. 32, 201-210.

Bechambi, O., Touati, A., Sayadi, S., Najjar, W., 2015c. Effect of cerium doping on the textural, structural and optical properties of zinc oxide: Role of cerium and hydrogen peroxide to enhance the photocatalytic degradation of endocrine disrupting compounds. Mat. Sci. Semicon. Proc. 39, 807-816.

Diao, Z.-H., Wei, Q., Guo, P.-R., Kong, L.-J., Pu, S.-Y., 2018. Photo-assisted degradation of bisphenol A by a novel FeS_2_@SiO_2_ microspheres activated persulphate process: Synergistic effect, pathway and mechanism. Chem. Eng. J. 349, 683-693.

Gao, S., Guo, C., Lv, J., Wang, Q., Zhang, Y., Hou, S., Gao, J., Xu, J., 2017. A novel 3D hollow magnetic Fe_3_O_4_/BiOI heterojunction with enhanced photocatalytic performance for bisphenol A degradation. Chem. Eng. J. 307, 1055-1065.

Jasso-Salcedo, A.B., Palestino, G., Escobar-Barrios, V.A., 2014. Effect of Ag, pH, and time on the preparation of Ag-functionalized zinc oxide nanoagglomerates as photocatalysts. J. Catal. 318, 170-178.

Kamaraj, M., Ranjith, K.S., Rajeshwari, S., Rajendra, K.R.T., Hasna, A.S., 2014. Photocatalytic degradation of endocrine disruptor Bisphenol-A in the presence of prepared Ce_x_Zn_1-x_O nanocomposites under irradiation of sunlight. J. Environ. Sci. 26, 2362-2368.

Li, W., Wu, P.-x., Zhu, Y., Huang, Z.-j., Lu, Y.-h., Li, Y.-w., Dang, Z., Zhu, N.-w., 2015. Catalytic degradation of bisphenol A by CoMnAl mixed metal oxides catalyzed peroxymonosulfate: Performance and mechanism. Chem. Eng. J. 279, 93-102.

Li, X., Wang, Z., Zhang, B., Rykov, A.I., Ahmed, M.A., Wang, J., 2016. Fe_x_Co_3−x_O_4_ nanocages derived from nanoscale metal–organic frameworks for removal of bisphenol A by activation of peroxymonosulfate. Appl. Catal. B: Environ. 181, 788-799.

Miao, Y., Zhang, H., Yuan, S., Jiao, Z., Zhu, X., 2016. Preparation of flower-like ZnO architectures assembled with nanosheets for enhanced photocatalytic activity. J. Colloid Interf. Sci. 462, 9-18.

Peng, X., Tian, Y., Liu, S., Jia, X., 2017. Degradation of TBBPA and BPA from aqueous solution using organo-montmorillonite supported nanoscale zero-valent iron. Chem. Eng. J. 309, 717-724.

Qiu, Y., Yang, M., Fan, H., Xu, Y., Shao, Y., Yang, X., Yang, S., 2013. Synthesis and characterization of nitrogen doped ZnO tetrapods and application in photocatalytic degradation of organic pollutants under visible light. Mater. Lett. 99, 105-107.

Rachna, Rani, M., Shanker, U., 2019. Sunlight active ZnO@FeHCF nanocomposite for the degradation of bisphenol A and nonylphenol. J. Environ. Chem. Eng. 7, 103153.

Rani, M., Shanker, U., 2018. Insight in to the degradation of bisphenol A by doped ZnO@ZnHCF nanocubes: High photocatalytic performance. J. Colloid Interf. Sci. 530, 16-28.

Selvam, N.C.S., Vijaya, J.J., Kennedy, L.J., 2013. Comparative studies on influence of morphology and La doping on structural, optical, and photocatalytic properties of zinc oxide nanostructures. J. Colloid Interf. Sci. 407, 215-224.

Sharma, J., Mishra, I.M., Dionysiou, D.D., Kumar, V., 2015. Oxidative removal of Bisphenol A by UV-C/peroxymonosulfate (PMS): Kinetics, influence of co-existing chemicals and degradation pathway. Chem. Eng. J. 276, 193-204.

Tan, Y.H., Goh, P.S., Ismail, A.F., 2015. Development of photocatalytic coupled zinc–iron oxide nanoparticles via solution combustion for bisphenol-A removal. Int. Biodeter. Biodegr. 102, 346-352.

Xu, L., Yang, L., Johansson, E.M.J., Wang, Y., Jin, P., 2018. Photocatalytic activity and mechanism of bisphenol a removal over TiO_2-x_/rGO nanocomposite driven by visible light. Chem. Eng. J. 350, 1043-1055.

Zhang, X., Ding, Y., Tang, H., Han, X., Zhu, L., Wang, N., 2014. Degradation of bisphenol A by hydrogen peroxide activated with CuFeO_2_ microparticles as a heterogeneous Fenton-like catalyst: Efficiency, stability and mechanism. Chem. Eng. J. 236, 251-262.

Zhu, H., Li, Z., Yang, J., 2018. A novel composite hydrogel for adsorption and photocatalytic degradation of bisphenol A by visible light irradiation. Chem. Eng. J. 334, 1679-1690.
